# Predictive Value of the CURB-65, MuLBSTA, Pneumonia Severity Index Scores and Laboratory Parameters for In-Hospital Mortality in Patients with Diabetes Mellitus and COVID-19 Infection

**DOI:** 10.3390/jcm15145424

**Published:** 2026-07-10

**Authors:** Andreea Magdalena Ghibu, Ionela Maniu, Victoria Birlutiu

**Affiliations:** 1 Faculty of Medicine, Lucian Blaga University of Sibiu, 550169 Sibiu, Romania; victoria.birlutiu@ulbsibiu.ro; 2 Infectious Diseases Department, Academic Emergency Hospital Sibiu, 550245 Sibiu, Romania; 3 Faculty of Sciences, Department of Mathematics and Informatics, Research Center in Informatics and Information Technology, Lucian Blaga University of Sibiu, 550024 Sibiu, Romania; 4 Research Team, Pediatric Clinical Hospital Sibiu, 550166 Sibiu, Romania

**Keywords:** COVID-19, diabetes mellitus, CURB-65, Pneumonia Severity Index, MuLBSTA

## Abstract

**Background/Objectives**: Several risk factors have been identified as being involved in the progression of COVID-19 over time. Among these, age, comorbidities, and various laboratory parameters have been reported. Diabetes mellitus is one of the most common comorbidities found among patients diagnosed with SARS-CoV-2 infection. Glucose metabolism dysfunction can exacerbate pre-existing diabetes or even induce diabetes mellitus. The purpose of this study is to assess the accuracy of the CURB-65, PSI, MuLBSTA severity scores and laboratory parameters in patients with diabetes mellitus diagnosed with COVID-19. **Methods**: We conducted a retrospective study involving patients with diabetes mellitus diagnosed with SARS-CoV-2 infection, performed between June 2023 and December 2024. **Results**: We analyzed 73 patients with pre-existing diabetes who were diagnosed with COVID-19. There were 56.16% men, living mostly in urban areas, with a median hospital stay of 9.38 days. Elevated levels of LDH (367.50, *p* = 0.002), CRP (133.90, *p* = 0.012), ferritin (724.25, *p* = 0.012), ESR (55.00, *p* = 0.014), D-dimers (2455.04, *p* = 0.004), white blood cells (12.76, *p* = 0.007) and the neutrophil-to-lymphocyte ratio (15.86, *p* = 0.021) were recorded in the group of deceased patients. Significantly elevated values were recorded for all three severity scores, the AUC for CURB-65 was 0.69, PSI 0.81 and MuLBSTA 0.86. **Conclusions**: Severity scores showed robust prognostic value in predicting mortality among patients with diabetes mellitus and COVID-19, emphasizing their relevance as clinically applicable tools for risk stratification and outcome assessment.

## 1. Introduction

The global impact of COVID-19 and the implications of this type of infection have led specialists in the field to identify and apply mortality prediction tools, whether related to in-hospital mortality or long-term mortality. Over time, various risk factors have been identified [1]. These include age or comorbidities, as well as laboratory parameters such as lymphopenia, the neutrophil-to-lymphocyte ratio, LDH, ferritin, or IL-6. Patients with diabetes mellitus represent a vulnerable group, susceptible to developing various types of infections, particularly among those with poor glycemic control [2,3]. The risk of developing complications of the disease is further amplified among patients who, in addition to this condition, have cardiovascular, renal or neurological disorders [4,5,6].

Tardive addressability and the onset of the cytokine storm have led to the development of critical forms of the disease, with significant pulmonary involvement, and have consequently been associated with increased mortality rates [7,8]. During this process, pro-inflammatory molecules are released. These include interleukins, specifically IL-1, IL-6, IL-8, IL-12, tumor necrosis factor-α, IFN-λ, IFN-β, and CXCL-10 [9,10]. Thus, acute respiratory distress syndrome develops, which is associated with other organ involvement as the condition progresses, leading to multiple organ failure and, consequently, death. Clinically, the patient presents with acute respiratory failure and significant auscultatory changes; from a laboratory perspective, there are increases in IL6, LDH, ferritin, and D-dimer levels, which translate into microvascular and macrovascular complications [11]. Furthermore, the glycemic alterations at admission are due to SARS-CoV-2 infection, through the action of the spike protein on pancreatic beta cells, causing their alteration or destruction [12]. Disruption of carbohydrate metabolism leads to the exacerbation of pre-existing diabetes or the induction of de novo diabetes mellitus, a phenomenon explained by the induction of insulin resistance. Thus, in clinical practice, increasing the amount of insulin administered to patients until the infectious process is controlled is essential for managing the chronic condition [13,14].

Based on these aspects and on the fact that diabetes mellitus is a chronic condition that makes the body more susceptible to infection, tools for predicting in-hospital mortality, which complete the medical care process, are necessary to maximize therapeutic outcomes. The use of such predictors assists clinicians in the early identification of cases with a potential risk of adverse outcomes or in-hospital death due to this type of infection, particularly within the aforementioned risk group [12,13].

The main objective of this study is to analyze admission laboratory parameters related to in-hospital mortality and determine the predictive accuracy at baseline of the CURB-65, MuLBSTA, and Pneumonia Severity Index scores among patients diagnosed with SARS-CoV-2 infection and prior diabetes.

## 2. Materials and Methods

### 2.1. Study Design and Participants

Data from patients diagnosed with SARS-CoV-2 infection at the Academic Emergency Hospital Sibiu between 1 January 2023, and 31 December 2024 were analyzed. These patients were tested using rapid antigen tests or RT-PCR. We analyzed patients hospitalized in the Infectious Diseases Clinic or those transferred to our clinic following a positive SARS-CoV-2 test. Patients with incomplete data and those transferred from other hospital clinics with incomplete medical histories or missing laboratory parameters were excluded. The present research includes 73 cases identified with known diabetes mellitus prior to admission. The data regarding the patient’s medical history of diabetes was extracted from the medical record. The study was conducted in accordance with the Declaration of Helsinki and with the approval of the ethics committees of Academic Emergency Hospital Sibiu (No. 32143/22 December 2021) and “Lucian Blaga” University of Sibiu (No. 26/16 June 2025).

### 2.2. Clinical and Laboratory Data

We collected detailed data related to patient age, place of origin, vaccination status, mode of ventilation, and status at discharge. We also recorded the patients’ main symptoms, including fever, chills, cough, dyspnea, headache, nasal congestion, rhinorrhea, myalgia, and arthralgia. Other respiratory symptoms were also analyzed, such as chest pain, the presence or absence of hemoptysis, cardiac symptoms (e.g., palpitations, arrhythmia, and precordial pain), gastrointestinal symptoms (nausea, vomiting, diarrhea, and abdominal pain), and the presence of asthenia and fatigue. Furthermore, associated comorbidities were analyzed, namely the presence of hypertension, nutritional status based on body mass index, the presence of chronic kidney disease, neurological conditions or malignancy. The presence of autoimmune, digestive, or urological conditions were also recorded.

In terms of laboratory tests, patients underwent imaging studies via chest X-ray or computed tomography to quantify the extent of lung involvement. Biologically, renal function, blood glucose levels upon admission, electrolyte levels, liver function, and inflammatory markers (including C-reactive protein, fibrinogen, and erythrocyte sedimentation rate) were assessed. Additionally, coagulation was assessed, including D-dimer and fibrin monomer levels, complete blood count and ferritin levels. The initiation of antiviral therapy was also recorded, as was the need for corticosteroid therapy or antibiotic therapy.

### 2.3. Severity Scores

Three severity scores were used, at baseline, in their original form: CURB-65, MuLBSTA, and the Pneumonia Severity Index. The CURB-65 score is an easy-to-use tool that quantifies five simple parameters. The score obtained guides clinicians regarding the need for hospitalization of patients with pneumonia, as follows: 0–1 point indicate that the patient can be treated on an outpatient basis; a score of 2 implies short-term monitoring; and a score greater than 3 indicates that the patient requires continuous hospitalization and monitoring (Appendix A Table A1) [15,16].

The MuLBSTA score, introduced during the pandemic, classifies viral pneumonia based on six simple parameters. These include lymphocyte count, age over 60, as well as the presence of bacterial co-infections, history of hypertension, smoking status, and lung involvement (Appendix A Table A1) [16,17].

The Pneumonia Severity Index adds complexity to patient assessment by combining clinical, laboratory, and imaging parameters and stratifying patients into five risk groups. The first risk group includes patients under 50 years of age, with or without comorbidities, who have a low risk of developing a severe form of the disease. The second group comprises patients with a score below 70 points, meaning that the first two groups allow for outpatient treatment. Risk group three corresponds to a score between 71 and 90. These patients require brief medical monitoring and are also associated with a low risk. Risk group IV corresponds to a score between 91 and 130, associated with moderate risk, while group V has a score above 130 points and corresponds to high risk (Appendix A Table A1) [16,18].

Upon completion of the selection process, patient data and the parameters of each score used were input into an Excel database, including the classification into risk groups specific to each score.

### 2.4. Statistical Analysis

Data are reported by mean and SD (standard deviation) or median and IQR (interquartile range) in the case of continuous variables and frequency and percentages in the case of categorical variables. Comparison between survivors and non-survivors groups was performed using Chi-square, Fischer’s and Mann–Whitney tests. Significant differences were considered at *p* < 0.05. To account for multiple testing across variables, *p*-values were adjusted using the Benjamini–Hochberg False Discovery Rate (FDR) procedure. Classification and Regression Tree (CART), a non-parametric, supervised machine learning method (that predicts target variables by evaluating interconnected predictors) was used to determine multilevel interactions among predictor variables and patient risk groups. ROC (Receiver Operator Characteristic) curves were used to assessed the discrimination ability of severity scores CURB-65, Pneumonia Severity Index and MuLBSTA. The cut-off value (corresponding to the largest Youden index = sensitivity + specificity − 1) and its sensitivity and specificity were determined. DeLong’s test for paired ROC curves was performed to evaluate whether the differences between the AUC values are statistically significant. Data analyses were performed using R software (v. 4.5.2., R Core Team, R Foundation for Statistical Computing, Vienna, Austria).

## 3. Results

### 3.1. Baseline Characteristics of Diabetic COVID-19 Patients

Of the 73 patients with diabetes mellitus included in the study, 56.16% were male, with a mean age of 73.23 ± 9.14, mainly from urban areas. There were 49 (67.12%) patients diagnosed with SARS-CoV-2 infection and pre-existing diabetes who were vaccinated, with two (56.16%) or three doses (39.72%). In addition to diabetes, the patients under study had at least one additional comorbidity, including cardiovascular, neurological, renal, or malignant conditions. Obesity was documented in 46.58% of the patients. In the study group, 62 patients (84.93%) survived, and 11 died (15.07%); the mean age in the survivors group was 73.47 ± 9.45, while in the deceased group it was 71.91 ± 7.35. The mean length of hospital stay was 9.38 ± 6.70 days; patients who died had significant longer hospital stay (14.36 ± 10.60 vs. 8.50 ± 5.41, *p* = 0.010), were more frequently admitted to ICU (72.73% vs. 6.45%, *p* < 0.001), and needed mechanical ventilation (54.55% vs. 1.61%, *p* < 0.001) (Table 1).

### 3.2. Laboratory Test Results

Laboratory characteristics are presented in Table 2. In the group of deceased patients, elevated levels of LDH (367.50 vs. 224.00, *p* = 0.002), CRP (133.90 vs. 61.41, *p* = 0.012), ferritin (724.25 vs. 279.80, *p* = 0.012), ESR (55.00 vs. 25.50, *p* = 0.014), and CPK (171.00 vs. 82.00, *p* = 0.700) were observed compared to the survivors. Also, on admission, levels of D-dimers (2455.04 vs. 855.03, *p* = 0.004), white blood cells (12.76 vs. 8.49, *p* = 0.007) and the neutrophil-to-lymphocyte ratio (15.86 vs. 4.94, *p* = 0.021) were higher in the group of deceased patients compared to survivors (Table 2).

### 3.3. Scores CURB-65, MuLBSTA and Pneumonia Severity Index

**Figure 1:**
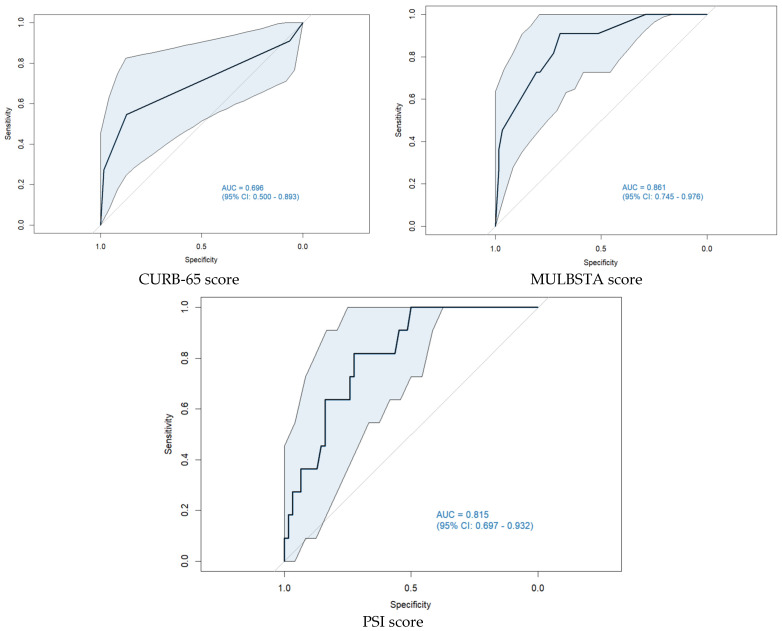
Receiver-operating characteristics (ROC) analysis.

Table 3 presents the comparison of severity scores between patients who died and survivors. For all scores, values are significantly higher in the group of patients who died in comparison with survivors (*p* < 0.001). ROC curves were generated to analyze the predictive value of these scores for the mortality. The AUC of CURB-65 was 0.69 (95% CI: 0.49–0.89), PSI 0.81 (95% CI: 0.69–0.93) and MuLBSTA 0.86 (95% CI: 0.74–0.97) (Table 3, Figure 1). DeLong’s test results revealed that the scores perform comparably (MuLBSTA vs. CURB: *p* = 0.230, MuLBSTA vs. PSI: *p* = 0.611, CURB vs. PSI: *p* = 0.221).

### 3.4. Decision Tree Analysis

**Figure 2:**
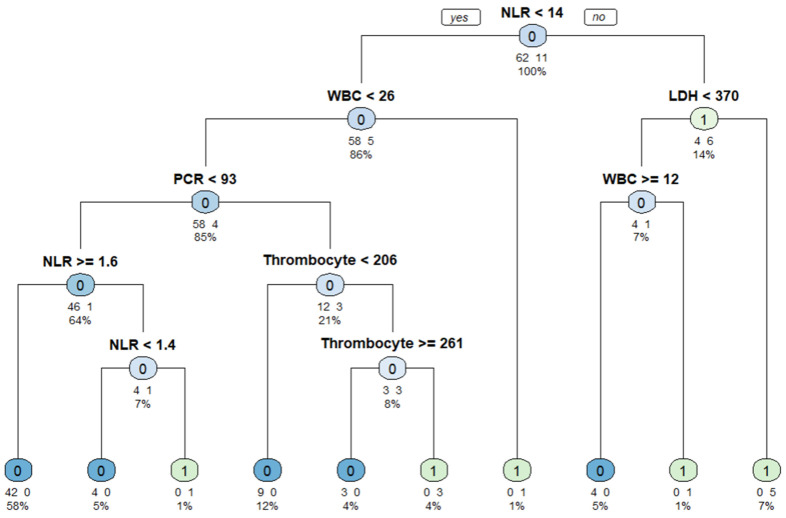
CART decision tree model.

The CART decision tree was constructed to stratify patient mortality risk based on multilevel interactions among predictor variables (Figure 1). The root split identified NLR, with threshold of 14, as the primary discriminator of mortality. For the risk group with NLR ≥ 14, LDH served as the secondary splitter, while patients exhibiting an LDH ≥ 370 demonstrated the highest severity profile, resulting in a 100% mortality rate (representing 7% of the total cohort, *n* = 5 deaths—high-risk group). Conversely, patients with NLR ≥ 14 but an LDH < 370 had a lower mortality risk. For the group with NLR < 14 (comprising 86% of the total sample), WBC count and PCR provided further stratification. The moderate-risk group had NLR < 14, WBC < 26 and PCR ≥ 93 (representing 4% of the total cohort, *n* = 3 deaths). The lowest-risk group was characterized by NLR < 14, WBC < 26, PCR < 93, yielding a 100% survival rate (*n* = 42, representing 58% of the entire study population). Patients from the high-risk group had higher score values when compared with low-risk group (MulBSA: 14.00 vs. 8.00, *p* < 0.001, PSI: 122 vs. 101, *p* = 0.034, CURB ≥ 3: 40% vs. 14.9%, *p* = 0.200) (Figure 2).

## 4. Discussion

The COVID-19 pandemic had a significant impact in patients with chronic conditions. Among those with diabetes, the pandemic led to poor control of blood glucose levels, with significant imbalances. Furthermore, there has been an increase in the number of diabetes cases, either due to patients with prediabetes progressing to diabetes or the emergence of new cases [19]. The literature has highlighted an inverse correlation between SARS-CoV-2 infection and diabetes, emphasizing that poor diabetes control and the development of microvascular complications have led to severe forms of COVID-19, prolonged hospitalizations, and difficult recoveries [20,21]. In addition, the use of corticosteroid therapy in severe/critical cases caused the onset of corticosteroid-induced diabetes. This has also contributed to increased mortality and morbidity.

This study is an observational, retrospective, single-center study involving 73 patients with COVID-19 and diabetes who were hospitalized in the Infectious Diseases Department of Academic Emergency Hospital Sibiu, Romania. In this study, we analyzed the general and laboratory characteristics of the patients at admission, in-hospital mortality, and the predictive value of the CURB-65, PSI, and MuLBSTA severity scores.

Among these patients, those who died had a mean age of 71.91 ± 7.35, comprised mainly men, and had a median length of hospital stay of 14.36 ± 10.60 days. Furthermore, a mortality rate of 15.06% was observed in this cohort, in line with the data reported in the literature [22,23,24]. Patients with diabetes had a higher body mass index, a finding that had previously been confirmed in a British population [22,25,26]. We report a higher prevalence of hypertension (79.45%) in those patients [27], followed by neurological disorders (32.88%) and malignancies (19.18%).

In the literature, the term “diabetic lung” has been used to describe changes in lung volumes and diffusion capacity attributed to diabetes mellitus and blood glucose levels above the normal range [28,29]. Consequently, in COVID-19 infection, uncontrolled blood glucose levels observed at the time of patient hospitalization caused significant pulmonary damage through the process of subclinical, subadacent pulmonary remodeling [28,30,31]. This is possible through metabolic dysfunction associated with diabetes, the presence of obesity, and multisystemic inflammation, as well as coagulopathy [32]. Consequently, cardiovascular events among diabetics are much more frequent, and consequently, mortality rates are higher [33]. Antiviral therapy and vaccination did not significantly influence mortality rates, as a large number of patients did not opt for vaccination. This was observed predominantly among the elderly, which means that this population group remains at risk of developing severe forms of the disease [34].

Of the patients with diabetes mellitus and COVID-19 infection, 59 patients had elevated blood glucose levels upon admission (>110 mg/dL). The SARS-CoV-2 virus targets ACE2 receptors in the pancreas, leading to impaired islet cell function. Due to impaired production by pancreatic β-cells, insulin secretion is reduced, and patients will have blood glucose levels above the normal range [35]. Furthermore, in the study cohort, deceased patients with diabetes mellitus exhibited elevated C-reactive protein and erythrocyte sedimentation rate values at admission, suggesting a more pronounced baseline inflammatory response. These findings may reflect an association between diabetes mellitus, systemic inflammation, and the development of severe or critical forms of COVID-19, in accordance with previously reported data in the literature [36]. Diabetic patients express higher levels of ACE2 in the lungs, which promotes the binding of the virus to pneumocytes, leading to severe lung damage. The cytokine storm, which frequently occurs in the second week of the disease, leads to organ dysfunction, with laboratory findings showing elevated serum levels of IL-6 [37], ferritin, and LDH, in addition to CRP levels [38,39,40,41]. This was also confirmed in our analysis.

In a study of 505 patients, including 136 with diabetes, Iqbal et al. concluded that an APTT > 24 s at admission, with or without anticoagulation, and a neutrophil-to-lymphocyte ratio > 8, were associated with higher mortality rates among patients with diabetes and COVID-19 [22]. In our study, slightly higher APTT values and significantly higher NLR values were observed in the group of patients with diabetes who died. This NLR ratio indicates a higher neutrophil count and relative lymphopenia, since diabetes causes immune system dysfunction, findings consistent with the inflammation underlying organ dysfunction [11]. No statistical differences were observed regarding lymphocyte count; however, the data are comparable to those previously published in an Italian and British population [22,42]. Furthermore, in the group of patients with diabetes who died, a higher number of leukocytes and neutrophils was observed, which was statistically significant and concordant correlated with the literature [4,19,43]. Anemia is also a common complication among patients with diabetes mellitus [20,22]. Low mean hemoglobin levels were also observed in our study, predominantly in the group of patients with diabetes and SARS-CoV-2 infection who died. The decision tree model revealed a relationship among inflammatory and tissue damage biomarkers in predicting patient mortality. The CART algorithm selected NLR as an important predictor, establishing an optimal clinical cut-off at 14, underscoring that severe systemic inflammation (indicated by elevated NLR) is a critical gateway to poor clinical outcomes. Furthermore, the model highlights a synergy between high systemic inflammation (NLR ≥ 14) and cellular tissue damage (LDH ≥ 370). When both thresholds are breached, mortality in our cohort reached 100%, suggesting that this specific biomarker combination could serve as a tool for early clinical triage. Conversely, the model identified a large group of patients (NLR < 14, WBC < 26, PCR < 93), representing over half of the cohort where mortality was absent.

The literature identifies various severity scores as good predictors of mortality in COVID-19 infection [16]. Our study focused on three of them, CURB-65, Pneumonia Severity Index, and MuLBSTA.

The CURB-65 score has been proved to be a predictor of mortality in patients with SARS-CoV-2 infection aged over 70 years at a score of 3, with a specificity of 83.2% but a sensitivity of 55.5%, since it is a score that does not account for patients’ comorbidities [37,44]. Diabetes mellitus is an important risk factor, reported in the literature as the seventh leading cause of death worldwide [34]. In our study, the results were AUC of 0.69 for this score. Similar results were obtained by Ion et al. in a study that assessed the usefulness of the APACHE, CURB, SOFA, and NEWS2 scores in predicting the severity of SARS-CoV-2 infection in patients with diabetes mellitus [45].

The Pneumonia Severity Index is a complex severity score that considers two important risk factors of COVID-19 infection: age and blood glucose levels. The high predictive value of this score has been demonstrated in several studies. One of these was conducted by Artero et al. in a comparative analysis of the CURB-65, PSI, MuLBSTA, and qSOFA scores, which highlights the superior predictive value of the PSI [46]. Similar results were also highlighted by Felippe Lazar Neto et al., achieving a sensitivity of 0.90 (95% CI: [0.86–0.93]) and an AUC of 0.79 (95% CI: [0.77–0.82]) [47,48].

The MuLBSTA is a score that emphasizes three important risk factors—smoking status, pulmonary involvement, and the presence of co-infections—being a good predictor of 90-day mortality [17]. In our group of patients with diabetes, higher mortality rates were observed with an average score of 13.45 (±2.98)/13.00 (11.00, 17.00), which is consistent with the literature at a cut-off point of ≥12 [49,50].

There are, however, several limitations that should be noted. First, this is a retrospective study conducted in a single centre with a small number of patients with SARS-CoV-2 infection and a prior diagnostic of diabetes (a specific subgroup of patients) and a limited number of deaths. The small number of outcome events limits the statistical power of our comparisons between survivors and non-survivors, accounting for the wide 95% confidence intervals for the AUCs. Consequently, the results should be considered exploratory and interpreted with caution. Another limitation is the fact that it is not possible to measure HbA1c as a matter of routine to monitor and assess glycemic control or to evaluate all patients for metabolic imbalances. The missing values for these parameters used to assess the severity of COVID-19 infection among diabetic patients limited our ability to provide a comprehensive overview of their predictive value. Furthermore, the dynamic assessment of diabetic patients to achieve glycemic control, as well as performing a comparative analysis regarding the prognosis of patients with diabetes versus those with corticosteroid-induced diabetes in COVID-19, represent future directions to be explored.

## 5. Conclusions

Diabetes patients belong to a vulnerable population group and require careful glycemic monitoring, especially against the backdrop of an infection. Glycemic control is a reliable solution to prevent unfavorable outcomes in patients with SARS-CoV-2 infection and diabetes. In our study, CURB-65, MuLBSTA, and Pneumonia Severity Index scores values were significantly higher in the group of patients who died in comparison with survivors. Integrating them into current practice could provide complexity to medical care, and their continuous assessment could offer flexibility and dynamism in the patient management process, both at admission and throughout hospitalization. Furthermore, validating these tools in large, multicenter studies in order to establish the accuracy of clinical cut-off points could lead to their use as important components for stratifying the risk of death among patients with diabetes and beyond.

## Figures and Tables

**Table 1:** Characteristics of patients.

Characteristics		All (*n* = 73)	Survivors (*n* = 62)	Non-Survivors (*n* = 11)	*p*-Value
Gender	M	41 (56.16%)	33 (53.23%)	8 (72.73%)	0.300
	F	32 (43.84%)	29 (46.77%)	3 (27.27%)	
Age	mean ± SD	73.23 ± 9.14	73.47 ± 9.45	71.91 ± 7.35	0.200
Environment	U	55 (75.34%)	45 (72.58%)	10 (90.91%)	0.300
	R	18 (24.66%)	17 (27.42%)	1 (9.09%)	
COVID-19 vaccination	Yes	49 (67.12%)	41 (66.13%)	8 (72.73%)	>0.900
	No	24 (32.88%)	21 (33.87%)	3 (27.27%)	
Weight	Normal	23 (31.51%)	18 (29.03%)	5 (45.45%)	0.300
	Overweight	16 (21.92%)	13 (20.97%)	3 (27.27%)	
	Obesity	34 (46.58%)	31 (50.00%)	3 (27.27%)	
Hypertension	Yes	58 (79.45%)	50 (80.65%)	8 (72.73%)	0.700
Chronic kidney disease	Yes	10 (13.70%)	8 (12.90%)	2 (18.18%)	0.600
Prior neurological condition	Yes	24 (32.88%)	20 (32.26%)	4 (36.36%)	>0.900
Tumor pathology	Yes	14 (19.18%)	10 (16.13%)	4 (36.36%)	0.200
ICU admission	Yes	12 (16.44%)	4 (6.45%)	8 (72.73%)	<0.001
Mechanic ventilation	Yes	7 (9.59%)	1 (1.61%)	6 (54.55%)	<0.001
Hospitalization days	mean ± SD	9.38 ± 6.70	8.50 ± 5.41	14.36 ± 10.60	0.010

**Table 2:** Tendencies of laboratory tests.

Characteristics	All (*n* = 73)	Survivors (*n* = 62)	Non-Survivors (*n* = 11)	*p*-Value/FDR adj. q-Value
Glicemia	148.00 (117.00, 189.00)	148.00 (119.00, 209.00)	137.00 (105.00, 154.00)	0.200/0.305
Urea	46.00 (34.00, 67.00)	46.00 (33.00, 67.00)	44.00 (39.00, 146.00)	0.700/0.725
Creatinine	1.06 (0.78, 1.53)	1.05 (0.78, 1.48)	1.23 (0.73, 1.99)	0.600/0.696
AST	27.00 (22.00, 38.00)	27.00 (22.00, 36.00)	46.00 (22.00, 79.00)	0.037/0.097
ALT	20.00 (16.00, 39.00)	19.00 (16.00, 34.00)	39.00 (17.00, 48.00)	0.100/0.207
PTsec ^a^	13.35 (12.35, 14.65)	13.00 (12.30, 14.60)	13.50 (13.40, 15.40)	0.100/0.207
Prothrombin activity ^b^	76.80 (64.05, 86.05)	77.90 (64.10, 86.90)	73.20 (59.70, 75.80)	0.120/0.217
INR ^c^	1.15 (1.08, 1.28)	1.13 (1.07, 1.27)	1.18 (1.16, 1.34)	0.100/0.207
APTT ^d^	32.50 (28.70, 39.60)	32.25 (28.70, 39.60)	33.50 (29.10, 46.20)	0.500/0.659
Sodium	138.00 (134.00, 140.00)	138.00 (134.00, 140.00)	136.00 (133.00, 146.00)	>0.900
Potassium	4.07 (3.72, 4.59)	4.12 (3.76, 4.60)	3.76 (3.40, 4.15)	0.120/0.217
Ferritin ^e^	288.80 (123.40, 581.40)	279.80 (103.50, 449.00)	724.25 (351.40, 1410.15)	0.012/0.049
LDH ^f^	241.00 (179.00, 332.00)	224.00 (175.00, 304.00)	367.50 (267.00, 711.00)	0.002/0.049
CPK ^g^	82.50 (58.50, 174.00)	82.00 (59.00, 163.00)	171.00 (19.00, 276.00)	0.700/0.725
Amilaza	53.00 (41.00, 76.00)	54.00 (42.00, 74.00)	44.00 (29.00, 151.00)	0.600/0.696
CRP	67.81 (23.61, 127.99)	61.41 (21.73, 96.28)	133.90 (67.81, 286.14)	0.012/0.049
Fibrinogen	504.30 (383.50, 593.00)	504.30 (392.30, 589.20)	501.80 (383.50, 909.20)	0.600/0.696
ESR ^h^	28.00 (13.50, 47.00)	25.50 (13.00, 44.00)	55.00 (33.00, 77.50)	0.014/0.050
D-dimers ^i^	1027.37 (626.38, 2488.70)	855.03 (533.99, 2150.15)	2455.04 (1642.85, 4239.09)	0.004/0.049
WBC	8.97 (6.34, 12.72)	8.49 (6.21, 10.76)	12.76 (9.57, 18.70)	0.007/0.049
Hemoglobin	13.40 (11.25, 14.60)	13.40 (11.30, 14.60)	12.10 (10.10, 14.10)	0.200/0.305
MCV	87.00 (83.25, 90.75)	86.70 (83.60, 89.50)	90.40 (82.70, 93.10)	0.300/0.435
MCHC	33.85 (32.95, 34.70)	33.90 (33.30, 34.70)	33.30 (31.00, 34.50)	0.200/0.305
RDWSD	44.95 (42.00, 49.30)	44.40 (41.60, 48.00)	49.30 (44.90, 52.30)	0.010/0.049
RDWCV	14.40 (13.35, 15.15)	14.00 (13.20, 14.90)	14.80 (14.50, 16.30)	0.031/0.089
Thrombocytes	189.50 (147.50, 256.50)	186.00 (146.00, 249.00)	252.00 (152.00, 333.00)	0.500/0.659
Neutrophils	6.70 (4.42, 8.78)	6.62 (3.84, 8.27)	9.29 (6.54, 16.34)	0.007/0.049
Lymphocytes	1.16 (0.73, 1.76)	1.18 (0.74, 1.73)	1.03 (0.54, 2.21)	0.700/0.725
NLR	5.68 (2.91, 10.93)	4.94 (2.78, 10.30)	15.86 (5.28, 27.76)	0.021/0.067

FDR adj. q-value—statistical significance values after Benjamini–Hochberg False Discovery Rate (FDR) correction, a,b,c—data missing for 3 survivors patients, d—data missing for 5 survivors patients, e—data missing for 9 survivors patients and 2 non-survivor patients, f,g—data missing for 5 survivors patients and 1 non-survivor patient, h—data missing for 4 survivors patients and 2 non-survivor patients, i—data missing for 15 survivors patients and 1 non-survivor patient.

**Table 3:** Comparison of severity scores.

Characteristics	All (*n* = 73)	Survivors (*n* = 62)	Non-Survivors (*n* = 11)	*p*-Value	Cut-Off Se/Sp
CURB-65	2.18 ± 0.63 2.00 (2.00; 2.00)	2.08 ± 0.49 2.00 (2.00; 2.00)	2.73 ± 1.01 3.00 (2.00; 3.50)	0.000	2.5 54.54/87.09
PSI	111.73 ± 32.46 106.00 (88.00, 131.00)	106.15 ± 29.63 101.50 (86.00, 125.00)	143.18 ± 30.81 140.00 (118.00, 163.00)	0.000	117 81.81/72.58
MulBSTA	9.30 ± 3.69 9.00 (8.00, 13.00)	8.56 (±3.31) 8.00 (7.00, 11.00)	13.45 ± 2.98 13.00 (11.00, 17.00)	0.000	9.5 90.90/69.35

Se—sensitivity, Sp—specificity.

## Data Availability

Data are contained within the article.

## Abbreviations

The following abbreviations are used in this manuscript:

SARS-CoV-2	Severe acute respiratory syndrome coronavirus 2
COVID-19	Coronavirus disease 2019
RT-PCR	Real-Time Polymerase Chain Reaction
BMI	Body Mass Index
SBP	Systolic Blood Pressure
DBP	Diastolic Blood Pressure
ICU	Intensive Care Unit
PSI	Pneumonia Severity Index
LDH	Lactate dehydrogenase
CRP	C-reactive protein
CPK	Creatine phosphokinase
AST	Aspartate aminotransferase
ALT	Alanine aminotransferase
ESR	Sedimentation rate of erythrocytes
WBC	White Blood Cells
NLR	Neutrophil-to-lymphocyte ratio
HbA1c	Hemoglobin A1C
ACE2	Angiotensin-Converting Enzyme 2
SOFA	Sequential Organ Failure Assessment Score
APACHE	Acute Physiology and Chronic Health Evaluation
NEWS	National Early Warning Score
qSOFA	Quick Sequential Organ Failure Assessment Score

## Appendix A

**Table A1:** CURB-65, MuLBSTA, PSI.

Characteristics	Yes	CURB-65	MuLBSTA	PSI
Confusion	+1	•		•
Blood urea ≥ 19 mg/dL	+1	•		>30 mg/dL +20
		•		
Respiratory rate ≥ 30 breaths/min	+1	•		+20
SBP < 90 mmHg, DBP ≤ 60 mmHg	+1	•		+20
Age		≥65 years old +1	≥60 years old +2	•
Male				Years
Female				Years − 10
Nursing home resident				Years + 10
Multilobar involvement	+5		•	
Lymphocyte count ≤ 0.8 × 10^9^/L	+4		•	
Bacterial infection	+4		•	
Smoking status			•	
Active smoker	+3			
Prior smoker	+2			
History of hypertension	+2		•	
Tumor/history of tumors	+30			•
Liver disease	+20			•
Congestive heart failure	+10			•
Cerebrovascular disease	+10			•
Kidney disease	+10			•
Altered mental status	+20			•
Temperature < 35 °C sau > 40 °C	+15			•
Heart rate > 125 bpm	+10			•
Arterial pH < 7.35	+30			•
Sodium < 130 mmol/L	+20			•
Glucose > 250 mg/dL	+10			•
Hematocrit < 30%	+10			•
Partial pressure of oxygen < 60 mmHg	+10			•
Abnormalities on chest X-ray	+10			Pleural effusion
